# Abdominal Pain as the First Presentation of Severe Heart Failure With Reduced Ejection Fraction and Left Ventricular Thrombus in a Young Asymptomatic Patient: A Diagnostic Interception

**DOI:** 10.1002/ccr3.73699

**Published:** 2026-10-14

**Authors:** Maryam Othman Alarfaj, Bashayr Saleh Alsuwailem, Meataz Khaled Aljeezan, Baker Saud Alhlibi, Mubarak Maher Bu Haya, Abdulatif Adel Alnaaim, Muneera O. AlTaweel

**Affiliations:** ^1^ Department of Internal Medicine King Abdulaziz Hospital, MNGHA Al‐Ahsa Saudi Arabia; ^2^ Department of Cardiac Sciences King Abdulaziz Hospital, MNGHA Al‐Ahsa Saudi Arabia; ^3^ King Abdullah International Medical Research Center (KAIMRC), Eastern Region, MNGHA Saudi Arabia; ^4^ King Saud Bin Abdulaziz University for Health Sciences (KSAU‐HS), Ministry of National Guard Health Affairs (MNGHA) Al‐Ahsa Saudi Arabia

**Keywords:** asymptomatic cardiomyopathy, cardiac magnetic resonance imaging, congestive hepatopathy, diagnostic interception, heart failure with reduced ejection fraction, left ventricular thrombus, young adults

## Abstract

A young, physically active patient with no cardiac symptoms presented with abdominal pain and was found, through incidental findings of elevated BNP, cardiomegaly, and new ECG changes, to have severe HFrEF with a left ventricular apical thrombus. Early cardiac evaluation changed management and led to complete thrombus resolution.

## 1 Introduction

The association between heart failure and hepatobiliary manifestations is well established. The cardiohepatic interaction—primarily hemodynamic in mechanism—results from elevated central venous pressure transmitted to the hepatic and portal venous systems, producing congestive hepatopathy, gallbladder wall edema, pericholecystic fluid, and a cholestatic pattern of liver enzyme abnormalities [1, 2, 3]. These findings can closely resemble acute cholecystitis both clinically and radiologically, and a number of case reports have described HFrEF presenting in this manner, including in young patients [4, 5, 6]. Autonomic connections between the cardiac and biliary systems have also been described, with vagally mediated reflexes capable of producing cardiac manifestations from biliary stimulation; however, in the context of heart failure producing hepatobiliary findings, the dominant and best‐supported mechanism is hemodynamic.

What has received comparatively less attention is the clinical profile of the patient in whom this scenario is most consequential: a young, physically active individual with no prior cardiac history, no symptoms of heart failure, and severe ventricular dysfunction that has gone entirely unrecognized. In such patients, the abdominal presentation is not merely a diagnostic mimic—it is paradoxically the first clinical event to bring the patient to medical attention. Without it, the underlying cardiac disease, including its thromboembolic complications, might have remained undetected indefinitely.

We report the case of a 38‐year‐old asymptomatic male who presented with epigastric pain and was found, through stepwise recognition of extra‐abdominal incidental findings, to have newly diagnosed severe HFrEF with a left ventricular apical thrombus. Cardiac MRI further identified late gadolinium enhancement (LGE) in the basal inferior wall, raising the possibility of an embolic ischemic substrate in addition to the known hypertensive risk. The case is presented not to recount the cardiohepatic mimic, which is already documented [4, 5, 6], but to illustrate how systematic attention to incidental findings within an abdominal presentation can intercept clinically silent severe cardiac disease and its complications, and to highlight the value of advanced cardiac imaging in establishing the tissue characterization data that guides prognosis and management.

## 2 Case History/Examination

A 38‐year‐old Saudi male presented with a 2‐day history of epigastric pain, gradual in onset, colicky, moderate in intensity, aggravated by fatty meals, and associated with nausea without vomiting. He explicitly denied chest pain, dyspnea, palpitations, and exercise intolerance, and reported maintaining a high level of physical activity with regular gym exercise several times per week. His medical history was notable only for recently diagnosed hypertension that had not yet been treated. He denied use of anabolic steroids, performance‐enhancing substances, stimulants, alcohol, or illicit drugs. He had no prior cardiovascular history and no family history of cardiomyopathy.

On examination, the patient appeared comfortable and hemodynamically stable. Mild sinus tachycardia was the only cardiovascular finding; there were no murmurs, signs of fluid overload, or respiratory distress. Abdominal examination revealed no tenderness, guarding, or a positive Murphy's sign.

## 3 Methods (Differential Diagnosis, Investigations, and Treatment)

Initial investigations demonstrated a cholestatic pattern with markedly elevated alkaline phosphatase (382 U/L) and bilirubin (21.3 μmol/L), mild leukocytosis, and mildly elevated CRP (12.8 mg/L) (Table 1). Serial troponin measurements remained within normal limits. BNP was moderately elevated at 443.5 pg/mL. Abdominal ultrasonography demonstrated gallbladder wall thickening and mild perihepatic fluid without gallstones or sludge (Figure 1), initially raising suspicion for acute cholecystitis. An incidental right‐sided pleural effusion was noted on the same study. Chest radiography demonstrated cardiomegaly with a globular cardiac silhouette, most consistent with left ventricular dilation (Figure 2).

**TABLE 1:** Laboratory results at presentation and follow‐up.

Lab	Reference values	Presentation (Day 0)	Follow‐up (~10 weeks)
WBC (×10^9^/L)	4–11	11.80	9.27
CRP (mg/L)	< 5	12.8	Not repeated
Total bilirubin (μmol/L)	< 20.5	21.3	9.0
Alkaline phosphatase (U/L)	40–150	382	189
ALT (U/L)	< 45	61	24
AST (U/L)	< 40	46	22
CK‐MB (ng/mL)	0–5.2	0.5	0.3
Troponin I (pg/mL)	< 34.2	14.7	< 10.0
BNP (pg/mL)	< 28.9	443.5 ↑	32.3 ✓

*Note:* CRP was measured only at presentation; it was not repeated at follow‐up. ↑ denotes a value above the reference range, and ✓ denotes a value that had normalized at follow‐up.

**FIGURE 1:**
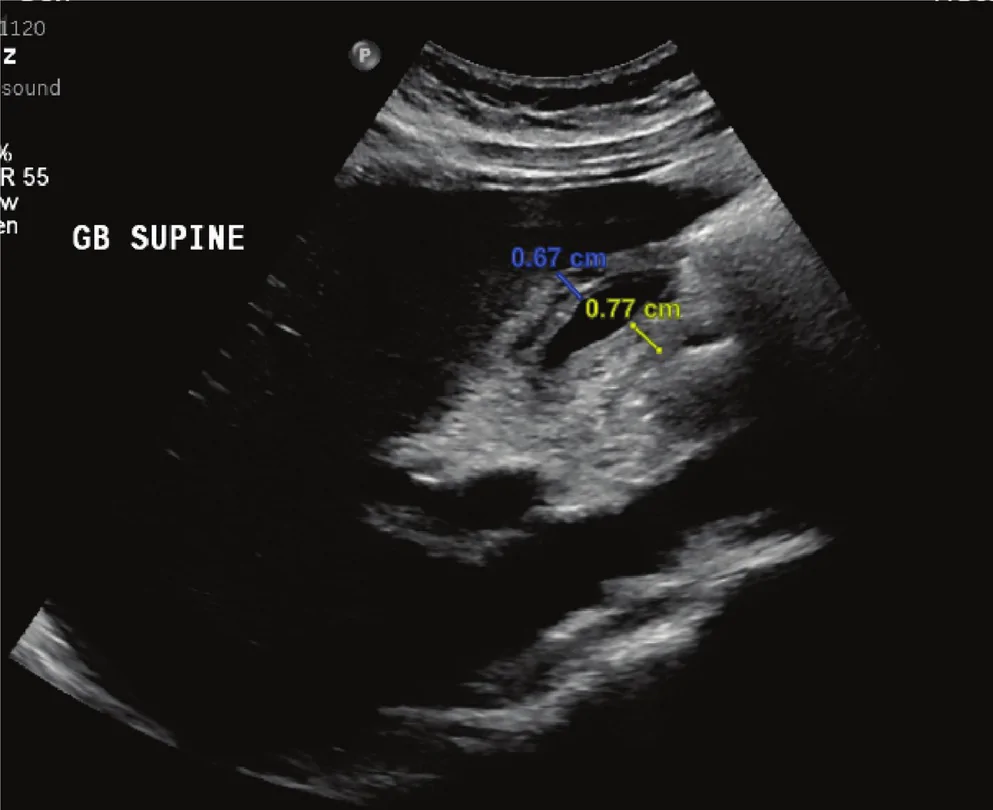
Abdominal ultrasonography demonstrating a contracted gallbladder with thickened, edematous wall (0.67–0.77 cm) and mild perihepatic free fluid. No gallstones or sludge were identified. An incidental right‐sided pleural effusion was also present on this study. These findings were attributed to congestive hepatopathy from severe HFrEF rather than primary biliary pathology.

**FIGURE 2:**
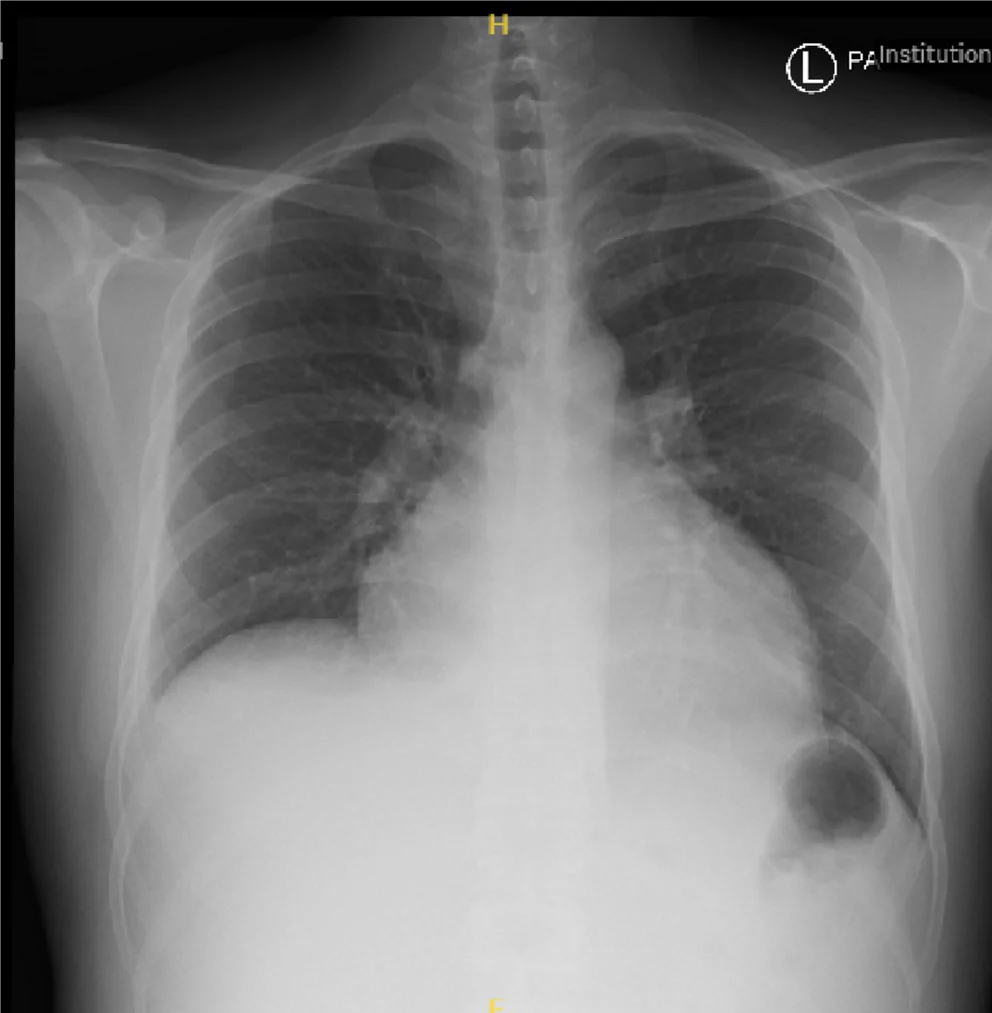
Chest radiograph demonstrating cardiomegaly with a globular cardiac silhouette, most consistent with left ventricular dilation confirmed on subsequent echocardiography. A small pericardial effusion was also identified on echocardiography, though its contribution to the cardiac contour on radiography cannot be excluded. This finding prompted urgent echocardiography.

Taken together—moderately elevated BNP, pleural effusion, and cardiomegaly in the absence of clinical evidence of infection—these findings prompted electrocardiography, which demonstrated new ST‐segment and T‐wave abnormalities in the lateral leads compared with prior recordings (Figures 3 and 4). The global rather than regional distribution of wall motion abnormality on subsequent echocardiography, combined with normal serial troponins, was considered inconsistent with an acute coronary syndrome. Urgent echocardiography revealed severely reduced left ventricular systolic function with an estimated ejection fraction of approximately 20% by M‐mode (Figure 5), severe global hypokinesia, a small pericardial effusion without tamponade, Grade I diastolic dysfunction with impaired LV relaxation, mild right ventricular systolic impairment, elevated filling pressures, and a dilated inferior vena cava of 3 cm—establishing the diagnosis of newly discovered HFrEF. Review of prior records revealed blood pressure readings as high as 188/130 mmHg; in the absence of another identified cause at this stage, markedly uncontrolled hypertension was considered the most likely contributor to the cardiomyopathy.

**FIGURE 3:**
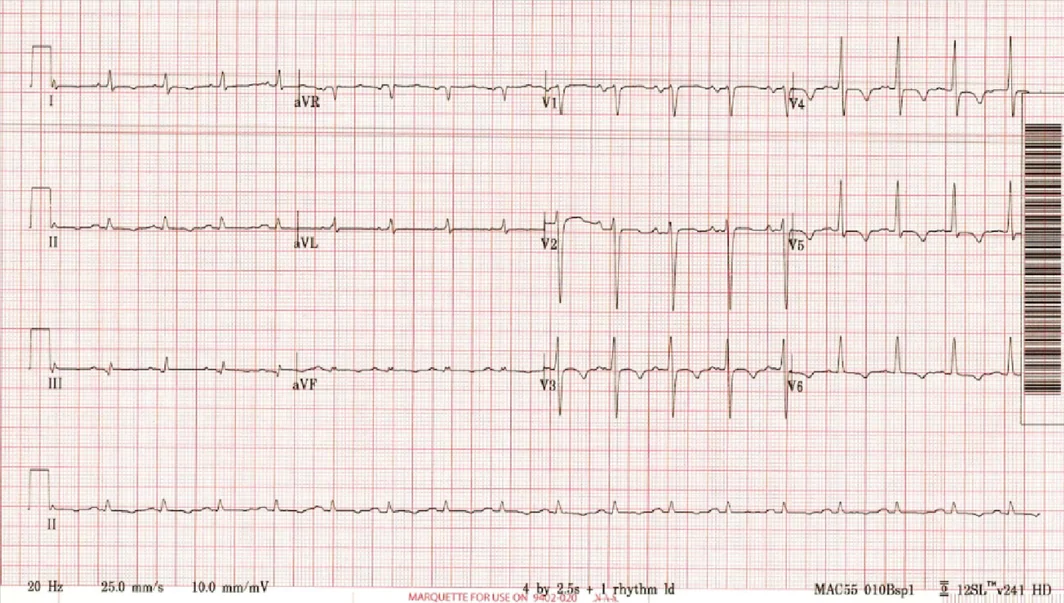
Electrocardiogram at presentation demonstrating sinus tachycardia with new lateral ST‐segment and T‐wave abnormalities, reported as abnormal with possible anterolateral ischemia. Serial troponin measurements remained within normal limits, and the global rather than regional wall motion pattern on echocardiography was considered inconsistent with acute coronary syndrome.

**FIGURE 4:**
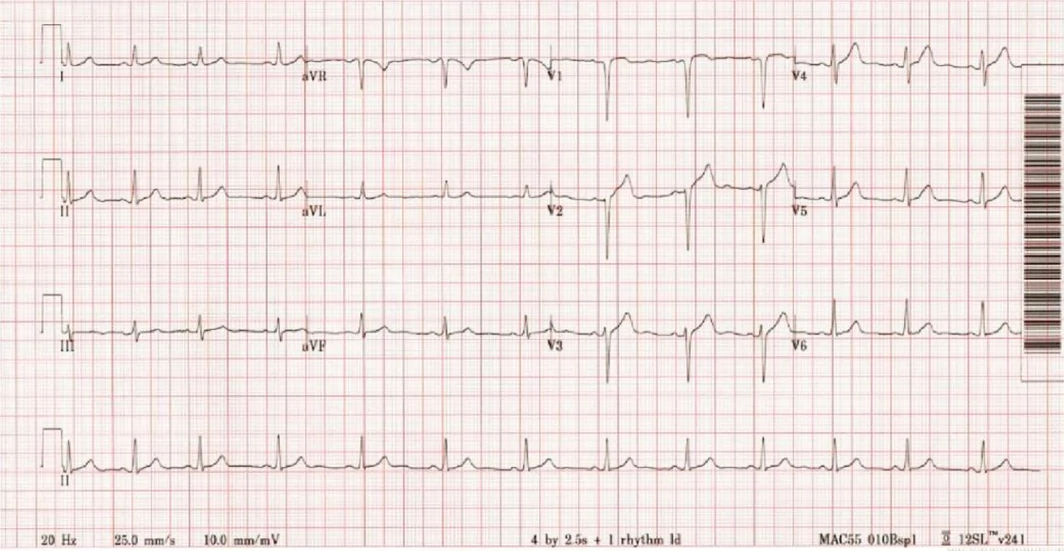
Prior baseline ECG demonstrating normal sinus rhythm without ST/T‐wave abnormalities, confirming the new onset of lateral changes at presentation.

**FIGURE 5:**
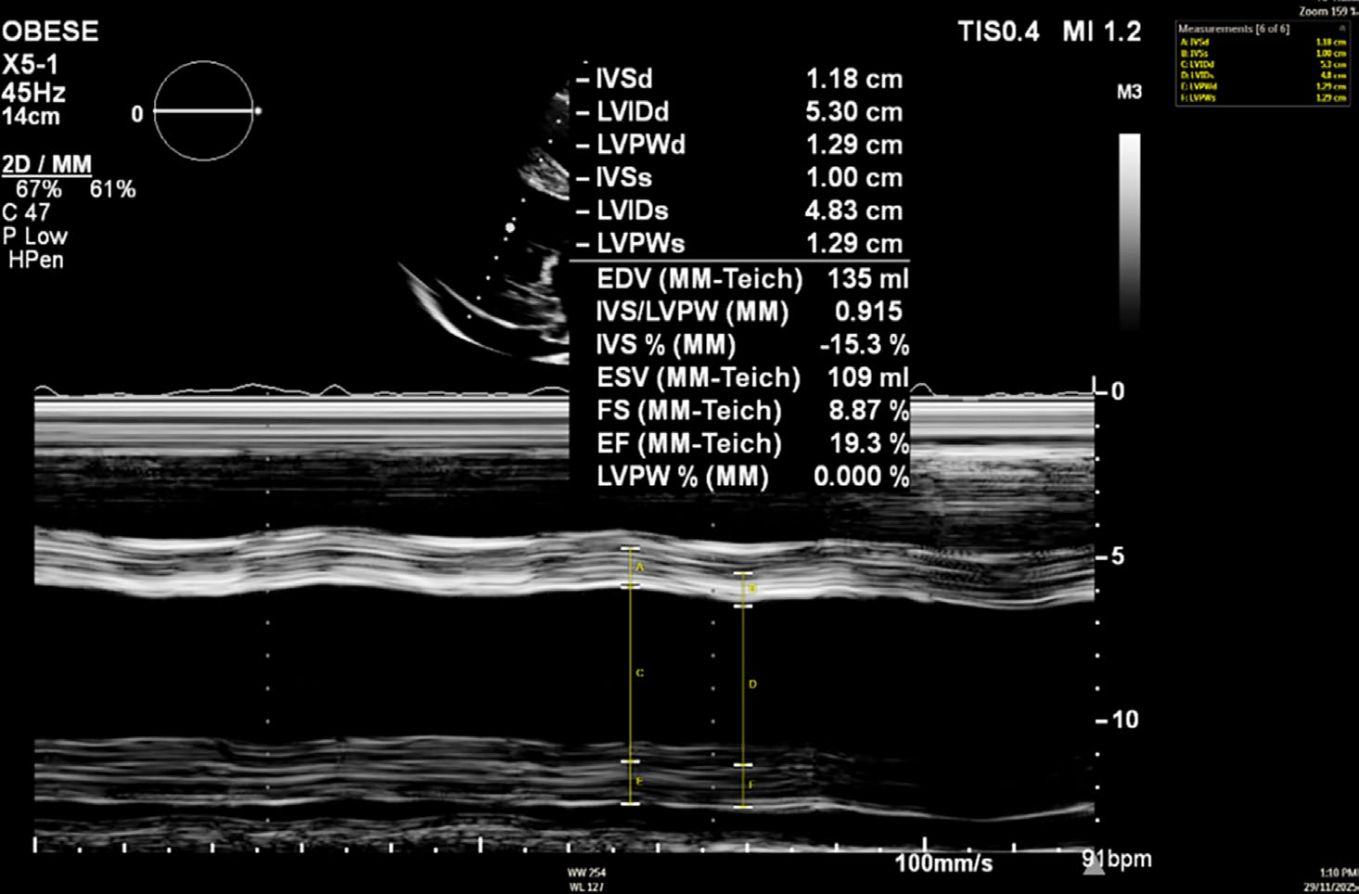
Transthoracic echocardiography (M‐mode) demonstrating severely reduced left ventricular systolic function with a measured ejection fraction of 19.3% by Teichholz method, fractional shortening of 8.87%, and markedly dilated left ventricular internal dimension in diastole (LVIDd 5.30 cm), consistent with newly diagnosed HFrEF.

The differential diagnosis for the presenting abdominal syndrome included acute calculous or acalculous cholecystitis, and this remained the leading clinical impression until the extra‐abdominal findings (pleural effusion, cardiomegaly, elevated BNP, new ECG changes) redirected the diagnostic pathway toward a cardiac etiology. The hepatobiliary findings were ultimately attributed to congestive hepatopathy; the absence of gallstones, negative Murphy's sign, afebrile state, and only mildly elevated inflammatory markers made acute infective cholecystitis unlikely. Surgical intervention was deferred [7, 8]. Guideline‐directed medical therapy was initiated, including bisoprolol 2.5 mg daily, candesartan, spironolactone 50 mg twice daily, and dapagliflozin 10 mg daily, and the patient was transferred to a tertiary cardiac center (KAMC‐R) for advanced evaluation.

At the tertiary center, cardiac magnetic resonance imaging (CMR) confirmed severe left ventricular systolic dysfunction (EF approximately 20%) and identified a left ventricular apical thrombus measuring 1.5 × 0.7 cm. LGE demonstrated a focus in the basal inferior wall consistent with a possible embolic ischemic substrate rather than a primary hypertensive or inflammatory pattern. Global myocardial native T1 time and extracellular volume fraction were normal, excluding significant diffuse fibrosis. There was no evidence of myocardial edema and no iron overload (T2* = 33 ms). The treating team considered the cardiomyopathy predominantly nonischemic in etiology, with the basal inferior LGE attributed to a possible embolic event in the context of the known apical thrombus; however, formal coronary angiography was not performed, and the etiology remains presumptive. Anticoagulation was initiated with heparin and transitioned to apixaban 5 mg twice daily. The patient was discharged on optimized guideline‐directed medical therapy including sacubitril/valsartan 200 mg twice daily, bisoprolol 5 mg daily, spironolactone 50 mg twice daily, dapagliflozin 10 mg daily, nifedipine extended‐release 60 mg daily, atorvastatin 40 mg daily, and apixaban 5 mg twice daily.

## 4 Conclusions and Results (Outcome and Follow‐Up)

At approximately 2 weeks, echocardiography demonstrated improvement in ejection fraction to 25%–30%, and abdominal symptoms had fully resolved without any biliary intervention. At approximately 10 weeks, ejection fraction had further improved to 38% on three‐dimensional (3D) echocardiography, BNP had normalized to 32.3 pg/mL (Table 1), and the apical thrombus had completely resolved on repeat imaging; anticoagulation was discontinued. The patient was transitioned to the general heart failure outpatient service. At 6‐month outpatient review (Dr. Gado, Cardiac Sciences), echocardiography demonstrated a mildly dilated left ventricle with moderate global hypokinesia, 3D LVEF 38%, Grade I diastolic dysfunction with normal filling pressures, mildly reduced right ventricular systolic function, and no pericardial effusion. The patient was clinically compensated on the same medication regimen and was planned for follow‐up in a further 5 months. The full clinical course is summarized in Table 2.

**TABLE 2:** Clinical timeline.

Timepoint	Events
Day 0 (presentation)	38‐year‐old male presents with 2‐day epigastric pain. Abdominal US: GB wall thickening, perihepatic fluid, right pleural effusion. CXR: cardiomegaly. BNP 443.5 pg/mL. New lateral ST/T changes on ECG. Echo: EF ~20%, severe global hypokinesia, IVC 3 cm
Days 1–3	Congestive hepatopathy diagnosed. Surgical intervention deferred. GDMT initiated (bisoprolol, candesartan, spironolactone, dapagliflozin). Transfer to tertiary cardiac center (KAMC‐R)
~Day 4–7 (tertiary center)	CMR: EF ~20%, LV apical thrombus 1.5 × 0.7 cm. LGE: basal inferior wall (possible coronary embolism); normal T1/ECV; no edema; no iron overload. Anticoagulation: heparin → apixaban 5 mg BID. Discharge on sacubitril/valsartan 200 mg BID, bisoprolol, spironolactone 50 mg, dapagliflozin 10 mg, nifedipine ER 60 mg, atorvastatin 40 mg, apixaban
~2 weeks	Echo: EF 25%–30%. Abdominal symptoms fully resolved
~10 weeks	Echo: EF 38%, 3D. BNP 32.3 pg/mL. Apical thrombus completely resolved. Apixaban discontinued. Transferred to general HF clinic
~6 months	Outpatient review (Dr. Gado). Echo: EF 38%, mildly dilated LV, moderate global hypokinesia, Grade I diastolic dysfunction, no pericardial effusion. Assessment: recovering dilated cardiomyopathy, compensated. Plan: continue GDMT, follow‐up in 5 months

## 5 Discussion

This case is not primarily about heart failure mimicking cholecystitis—that phenomenon is already well described in the literature, including in young patients [4, 5, 6], and a close precedent exists in which dilated cardiomyopathy presented through an abdominal and hepatobiliary syndrome [9]. The combination described here—clinically silent severe HFrEF, left ventricular apical thrombus, and abdominal presentation in a young asymptomatic patient with subsequent complete thrombus resolution and sustained ventricular recovery—has received limited attention in the existing case literature, and no report identified in our review described this specific combination with documented CMR tissue characterization as part of the clinical narrative.

### 5.1 Clinically Silent Severe HFrEF in a Young, Physically Active Patient

The patient presented with an ejection fraction of approximately 20%, severe global hypokinesia, a BNP of 443.5 pg/mL, and a dilated inferior vena cava of 3 cm—and yet reported no cardiac symptoms and was exercising regularly several times per week. This degree of functional compensation in the setting of such severe ventricular dysfunction reflects the capacity of young patients to upregulate compensatory mechanisms—sympathetic activation, peripheral vascular adaptation, and skeletal muscle efficiency—to a degree that masks even profoundly impaired cardiac function. This phenotype carries a specific clinical risk: the absence of typical symptoms that would otherwise prompt cardiac evaluation means that severe disease accumulates undetected. In this patient, the abdominal presentation was the clinical event that enabled diagnosis.

### 5.2 The Diagnostic Interception Sequence

The case illustrates how a chain of apparently incidental findings, each individually attributable to biliary disease, collectively pointed to a cardiac etiology and redirected management before an invasive biliary pathway was pursued. Abdominal pain and gallbladder wall thickening raised initial suspicion for acute cholecystitis. An incidental right‐sided pleural effusion on abdominal ultrasonography prompted chest radiography, which demonstrated cardiomegaly. A moderately elevated BNP of 443.5 pg/mL—not a recognized feature of isolated biliary pathology—combined with the imaging findings, prompted electrocardiography, which revealed new lateral ST/T‐wave changes. These triggered urgent echocardiography, which established the diagnosis of severe HFrEF. Surgical intervention was deferred, and the patient was transferred for advanced cardiac evaluation, at which point CMR identified the apical thrombus and provided tissue characterization data. At each step, a single finding that might have been set aside as incidental was instead recognized as part of a pattern inconsistent with isolated biliary pathology.

### 5.3 Left Ventricular Thrombus, CMR Findings, and Etiologic Uncertainty

Left ventricular thrombus is a recognized complication of HFrEF, occurring with higher frequency in patients with ejection fractions below 25% and apical wall motion abnormalities [10]. Its detection on CMR in this case changed management: anticoagulation was initiated, which would not have been pursued had the presentation been managed as biliary disease alone.

The CMR tissue characterization findings introduce important etiologic nuance. LGE in the basal inferior wall was identified, with a pattern considered consistent with a possible embolic ischemic substrate rather than the diffuse nonischemic fibrosis pattern typically associated with hypertensive or idiopathic dilated cardiomyopathy. Global native T1 time and extracellular volume fraction were normal, excluding significant diffuse fibrosis. The treating team at the tertiary center considered the cardiomyopathy predominantly nonischemic, attributing the basal inferior LGE to a possible embolic event in the context of the apical thrombus; however, formal coronary angiography was not performed. Other reversible and secondary causes—including myocarditis, infiltrative disease, tachycardia‐mediated cardiomyopathy, and toxic causes—were considered clinically unlikely given the absence of supporting features, the patient's denial of anabolic steroid or stimulant use, and the echocardiographic and CMR pattern. Thyroid function and metabolic workup were within normal limits. The etiology therefore remains presumptive, with uncontrolled hypertension as the most likely primary contributor in the context of a possible superimposed embolic event.

The favorable clinical trajectory—EF improving from 20% to 38%, BNP normalizing from 443.5 to 32.3 pg/mL, complete thrombus resolution, and maintained compensation at 6 months—is consistent with the responsiveness of nonischemic or hypertensive cardiomyopathy to guideline‐directed medical therapy when initiated before irreversible fibrosis occurs [11]. Whether this recovery reflects the underlying reversibility of the cardiomyopathy, the treatment effect, or both cannot be determined from this single case; the temporal association with therapy initiation is noted without implying direct causation.

### 5.4 Diagnostic Implications

In any patient presenting with abdominal pain and hepatobiliary findings, a moderately elevated BNP, unexplained cardiomegaly, or pleural effusion should prompt urgent echocardiography before surgical referral, regardless of age or functional status. When severe LV dysfunction is identified, cardiac MRI adds important diagnostic value beyond thrombus detection: tissue characterization by LGE, T1 mapping, and T2* can help differentiate ischemic from nonischemic substrate, identify inflammatory or infiltrative disease, and assess the extent of fibrosis—all of which bear on prognosis, likelihood of recovery, and management decisions. In this case, CMR‐guided management extended to anticoagulation for the apical thrombus and informed the discussion of coronary evaluation for the basal inferior LGE.

### 5.5 Conclusion

This case describes a young, physically active patient in whom clinically silent severe HFrEF with a left ventricular apical thrombus was discovered through an abdominal presentation. Systematic recognition of extra‐abdominal incidental findings redirected management, enabled the discovery and treatment of the thrombus, and led to its complete resolution alongside sustained ventricular recovery at 6 months. CMR tissue characterization added etiologic nuance—basal inferior LGE suggesting a possible embolic substrate—that would not have been available without advanced imaging. Clinicians encountering young patients with abdominal pain and unexplained systemic findings should maintain a low threshold for cardiac evaluation; in such patients, the abdominal presentation may be the only opportunity for diagnosis.

#### 5.5.1 Learning Points

- Severe HFrEF can remain clinically silent in young, physically active patients; an abdominal presentation may be the first—and only—reason the patient seeks medical attention, making it the unexpected gateway to cardiac diagnosis.
- A chain of subtle extra‐abdominal findings—moderately elevated BNP, unexplained cardiomegaly, and pleural effusion—should prompt urgent echocardiography even when the primary complaint is abdominal, regardless of age or functional status.
- Left ventricular thrombus is a recognized complication of severe HFrEF with ejection fractions below 25%; its identification changed management in this case before an invasive biliary pathway was pursued.
- Cardiac MRI provides important tissue characterization data beyond thrombus detection; in this case, LGE in the basal inferior wall raised the possibility of an embolic ischemic substrate, highlighting the value of advanced imaging in young patients with newly diagnosed dilated cardiomyopathy of uncertain etiology.

## Author Contributions

**Maryam Othman Alarfaj:** conceptualization, writing – original draft, writing – review and editing, data curation. **Bashayr Saleh Alsuwailem:** data curation, writing – original draft. **Meataz Khaled Aljeezan:** investigation, data curation, writing – review and editing. **Baker Saud Alhlibi:** investigation, data curation, writing – review and editing. **Mubarak Maher Bu Haya:** investigation, data curation, writing – original draft. **Abdulatif Adel Alnaaim:** investigation, data curation, writing – original draft. **Muneera O. AlTaweel:** conceptualization, writing – review and editing, supervision.

## Funding

The authors have nothing to report.

## Ethics Statement

Ethical approval was obtained from the IRB of King Abdullah International Medical Research Center (IRB approval no: NRA26/008/3).

## Consent

Written informed consent was obtained from the patient.

## Conflicts of Interest

The authors declare no conflicts of interest.

## Data Availability

The data that support the findings of this study are available on request from the corresponding author. The data are not publicly available due to privacy or ethical restrictions.
